# Identification and Characterization of microRNA319a and Its Putative Target Gene, *PvPCF5*, in the Bioenergy Grass Switchgrass (*Panicum virgatum*)

**DOI:** 10.3389/fpls.2017.00396

**Published:** 2017-03-30

**Authors:** Qi Xie, Xue Liu, Yinbing Zhang, Jinfu Tang, Dedong Yin, Bo Fan, Lihuang Zhu, Liebao Han, Guilong Song, Dayong Li

**Affiliations:** ^1^Institute of Turfgrass Science, College of Forestry, Beijing Forestry UniversityBeijing, China; ^2^State Key Laboratory of Plant Genomics and National Center for Plant Gene Research, Institute of Genetics and Developmental Biology, Chinese Academy of SciencesBeijing, China; ^3^CAS Key Laboratory of Genome Sciences and Information, Beijing Institute of Genomics, Chinese Academy of SciencesBeijing, China; ^4^State Key Laboratory Breeding Base of Dao-di Herbs, National Resource Center for Chinese Materia Medica, China Academy of Chinese Medical SciencesBeijing, China; ^5^ShenZhen Guo Yi Park Developments Co. LtdShenzhen, China

**Keywords:** switchgrass (*Panicum virgatum*), leaf development, microRNA319 (miR319), bioenergy, TCP transcription factor

## Abstract

Due to its high biomass yield, low environmental impact, and widespread adaptability to poor soils and harsh conditions, switchgrass (*Panicum virgatum* L.), a warm-region perennial herbaceous plant, has attracted much attention in recent years. However, little is known about microRNAs (miRNAs) and their functions in this bioenergy grass. Here, we identified and characterized a miRNA gene, *Pvi-MIR319a*, encoding microRNA319a in switchgrass. Transgenic rice lines generated by overexpressing the *Pvi-MIR319a* precursor gene exhibited broader leaves and delayed flowering compared with the control. Gene expression analysis indicated at least four putative target genes were downregulated. Additionally, we cloned a putative target gene (*PvPCF5*) of *Pvi-MIR319a* from switchgrass. PvPCF5, a TCP transcription factor, is a nuclear-localized protein with transactivation activity and control the development of leaf. Our results suggest that *Pvi-MIR319a* and its target genes may be used as potential genetic regulators for future switchgrass genetic improvement.

## Introduction

Switchgrass (*Panicum virgatum* L.), which is highly adaptable and particularly resistant to stress, can survive in many climatic conditions, including in a variety of soil conditions (Vogel et al., 2010). It is used in several bioenergy conversion processes, such as ethanol production, biogas, and thermal energy applications (McLaughlin and Adams Kszos, 2005). Generally, switchgrass can be grouped into two main ecotype categories: lowland, with tall height and high output, and upland, with short stature and low biomass output (Gunter et al., 1996). Switchgrass has been increasingly regarded as a model energy crop with huge potential in the United States and worldwide (McLaughlin and Adams Kszos, 2005; David and Ragauskas, 2010; Monti, 2012). Genetic engineering can improve plant traits and enhance stress tolerance (Hisano et al., 2011; Li and Qu, 2011). Application of transgenic biotechnologies and genomic analyses will contribute to the development of bioenergy using switchgrass (Sticklen, 2006; Bouton, 2007).

In plants, microRNAs (miRNAs), one of the prominent posttranscriptional regulators, are a class of ∼19–24 nucleotide (nt) endogenous non-coding small RNAs (Reinhart et al., 2002; Chen, 2008; Fabian et al., 2010; Liu and Chen, 2016). These small RNAs form an imperfect stem-loop structure in single-stranded primary miRNAs through recognition by a Dicer-like protein, DCL1, and mature miRNAs are recruited into an RNA-induced silencing complex (RISC) to control target gene expression by either direct mRNA cleavage or translational repression (Bartel, 2004). Recent evidence indicates that miRNAs are important genetic regulators and play critical roles in plant development, nutrient homeostasis, and various biotic and abiotic stress responses, including salinity, drought, and extreme temperatures, via interaction with their targets (Chen, 2008; Chuck et al., 2011; Zhou and Luo, 2013; Zhou et al., 2013; He et al., 2014).

Studies on switchgrass include examinations of the establishment and optimization of a transformation system (Li and Qu, 2011), gene expression analysis, and identification of miRNAs (Chuck et al., 2011; Fu et al., 2012; Li et al., 2014). For example, by utilizing bioinformatics and experimental approaches, Matts et al. (2010) identified ∼20 conserved miRNA families, predicted 37 miRNA target genes, and confirmed 4 target mRNAs using a modified 5′ RACE (Rapid Amplification of cDNA Ends) strategy. They also found that miR395 and miR399 have distinct expression patterns compared with other species, and this may be related to the wide adaptability of switchgrass growth. Furthermore, using a well-defined computational approach, Xie et al. (2010) identified 121 predicted miRNAs from 44 miRNA families and 839 potential targets in switchgrass. Transgenic switchgrass overexpressing miR156 exhibited different morphological phenotypes compared with wild-type, and the degree of alteration depended on miR156 levels, where relatively low miR156 levels could increase biomass yield (Chuck et al., 2011; Fu et al., 2012).

In plants, microRNA319 (miR319) is one of the most conserved and ancient microRNA families, which was found in diverse plant species from moss to flowering plants (Palatnik et al., 2003, 2007; Pandey and Baldwin, 2007; Cuperus et al., 2011). The miR319s have been demonstrated to target *TCP* (TEOSINTE BRANCHED/CYCLOIDEA/PROLIFERATING CELL FACTORS) genes, which encode plant-specific transcription factors (Nag et al., 2009; Martín-Trillo and Cubas, 2010). The TCP transcription factors have a conserved TCP domain with a basic helix-loop-helix structure and this family is known to play essential roles in diverse biological processes, including various aspects of plant growth and development, responses to biotic and abiotic stresses (Palatnik et al., 2003; Ori et al., 2007; Nag et al., 2009; Danisman, 2016).

The recent studies showed that overexpression of miR319 led to abnormal development and tolerance to stress in several transgenic plants (Yang et al., 2013; Zhou et al., 2013; Wang et al., 2014; He et al., 2014; Zhou and Luo, 2014; Li et al., 2016). High levels of Osa-miR319 in transgenic rice led to wider leaf blades and significantly enhanced cold tolerance in acclimated transgenic plants (Yang et al., 2013). Furthermore, other researchers suggested that osa-miR319b may play a role in the response to cold stress *via* control of *OsPCF6* and *OsTCP21* (Wang et al., 2014). In addition, Zhou et al. (2013) found that transgenic creeping bentgrass (*Agrostis stolonifera*) overexpressing *Osa-miR319a* displayed morphological changes in leaves and stems with a parallel increased tolerance to salinity and drought stress (Zhou et al., 2010, 2013; Yang et al., 2013). However, few studies have examined the roles played by several important microRNAs, such as miR319, in switchgrass. The genetic engineering and application of miRNAs provides an opportunity to make effective improvements in switchgrass (Zhou et al., 2013; Li et al., 2014).

In this study, we cloned and characterized *Pvi-MIR319a*, which encodes a microR319 in switchgrass. *Pvi-MIR319a*-overexpressing transgenic rice lines were used to investigate the role of miR319 in plant morphological development. We also isolated and identified a putative target gene (*PvPCF5*) of *Pvi-MIR319a* in switchgrass; this is a nuclear localization protein with transcriptional activation activity that is negatively regulated by *Pvi-MIR319a*. These data suggest that *Pvi-MIR319a* and its targets may possess important biological functions in switchgrass.

## Materials and Methods

### Plant Materials and Growth Conditions

Switchgrass (*P. virgatum* L.) cultivar Alamo was used in this study. The seeds were provided by Dr. Wanjun Zhang (Department of Grassland Science, China Agricultural University, Beijing, China). The switchgrass were planted in the greenhouse and the experimental station of the Institution of Genetics and Developmental Biology, Chinese Academy of Sciences (IGDB, CAS), China.

Rice (*Oryza sativa* L.) subspecies *japonica* cultivar Zhonghua 11 (ZH-11) was used as transgenic materials in this study. The transgenic and wild type control rice plants were grown in the greenhouse at 28°C for 14 h (day) and 10 h (night) and the field of experimental station.

### Cloning of the *Pvi-MIR319a* Gene, Construction of Plant Expression Vectors, and Rice Transformation

The *Osa-MIR319a* sequence (ID: MI0001098) was downloaded from the miRNA database^1^ (Kozomara and Griffiths-Jones, 2013). By searching with this sequence in the NCBI, we obtained the putative *Pvi-MIR319a* EST sequence from switchgrass.

To overexpress the switchgrass *Pvi-MIR319a* gene in rice, *Pvi-MIR319a* cDNA containing a stem-loop structure was cloned *via* RT-PCR amplification using the gene-specific primers 5′-TCTAGATTGAGTTTATGGCTTCTCTGGAAGA-3′ and 5′-GTCGACTGAGGTGTTCTATTGGTAGCCCAA -3′; the cloned DNA fragment was then inserted into the *Xba* I and *Sal* I sites of the binary vector pZH01, producing p35S-*Pvi-MIR319a*/p35S-hyg. The construct, containing the CaMV 35S promoter, was transferred into *Agrobacterium tumefaciens* strain LBA4404 by electroporation. Rice was transformed using the method of Hiei et al. (1994).

### Phylogenetic Analysis of TCPs

TCP (TEOSINTE BRANCHED1/CYCLOIDEA/PROLIFERATING CELL NUCLEAR ANTIGEN FACTOR) protein sequence data were downloaded from the Arabidopsis Information Resource^2^ and the MSU Rice Genome Annotation Project Database and Resource^3^, respectively. Multiple alignments of TCP protein family members from rice and arabidopsis were constructed using Clustal X (Wagner et al., 2008). A phylogenetic tree was constructed using the MEGA5 program (Tamura et al., 2011).

### RNA Isolation, cDNA Preparation, and Quantitative RT-PCR

Total RNA was extracted from switchgrass using TRIzol reagent (Invitrogen, Carlsbad, CA, USA) following the manufacturer’s protocol. Genomic DNA was removed using DNase I (Takara, Japan). For real-time PCR, synthesis of the first-strand cDNA was terminated by applying M-MLV reverse transcriptase (Promega, Madison, WI, USA). Genomic DNA was removed using DNase I (Takara, Japan). Quantitative RT-PCR was performed on a Bio-Rad Chromo 4 Real-Time PCR System (Bio-Rad, Hercules, CA, USA) using a TransStart SYBR Green qPCR SuperMix Kit (TransGen, Beijing, China). A switchgrass ubiquitin gene was used as a control (Wuddineh et al., 2015) (Switchgrass Unitranscript ID: AP13CTG25905). Expression levels of examined genes were quantified by a relative quantitation method, each sample was subjected to at least three biological replicates in this experiment. The specific primers used in this study were listed in **Supplementary Table S1**.

### *Pvi-MIR319a* and *PvPCF5* Transient Expression

To observe whether *PvPCF5* is a *Pvi-MIR319a* target gene, *Nicotiana benthamiana* leaves were used in this experiment (Llave and Carrington, 2002). The *PvPCF5* coding regions were inserted into an pEZR(K)-LC vector (LC) containing the CaMV35S promoter and green fluorescence protein (GFP) to generate 35S::PvPCF5-GFP (LC-PCF5), while the *Pvi-MIR319a* was inserted into the LC vector that lack GFP. We separated these into four groups: (a) LC-vector; (b) LC-PCF5; (c) LC-PCF5 and LC-319; and (d) LC-319. These four groups of plasmids were injected into 28-day-old tobacco leaves and observed 3 days later. The specific primers used in this study were listed in **Supplementary Table S1**.

### Subcellular Localization in Switchgrass

Young etiolated seedlings leaves were cut and digested with 20 ml of enzyme solution to prepare the protoplasts. The fusion gene (LC-PCF5) and the negative control pEZR(K)-LC vector were used for PEG (polyethylene glycol)-mediated transformation of switchgrass protoplasts (Liu et al., 2015). After 18 h of incubation, GFP fusion protein fluorescence signals were visualized using a fluorescence confocal microscope (Zeiss Axio Imager.Z2)^4^.

### Transactivation Assay in Yeast Cells

The complete ORF of *PvPCF5*, N-terminal putative activation domain (1–299 amino acids), and C-terminal area (300–439 amino acids) were amplified and digested with *Eco*R I and *Bam*H I and fused to the pGBKT7-GAL-4 vector. An empty pBD vector (pGBKT7) was used as a negative control. These constructs and the empty vector were each introduced into yeast strain Y2HGold containing the *AUR1-C* and *MEL1* reporter genes. Yeast cell transformation was carried out using the instructions in the Yeastmaker^TM^ Yeast Transformation System 2 User Manual^5^. The yeast transformants were selected on SD/-Trp and SD/-Trp/A/X-α-gel plates for 2–4 days at 30°C to identify transactivation activity (Yeast Protocols Handbook; Clontech, USA).

## Results and Discussion

### Identification and Characterization of *Pvi-MIR319a* in Switchgrass

**FIGURE 1:**
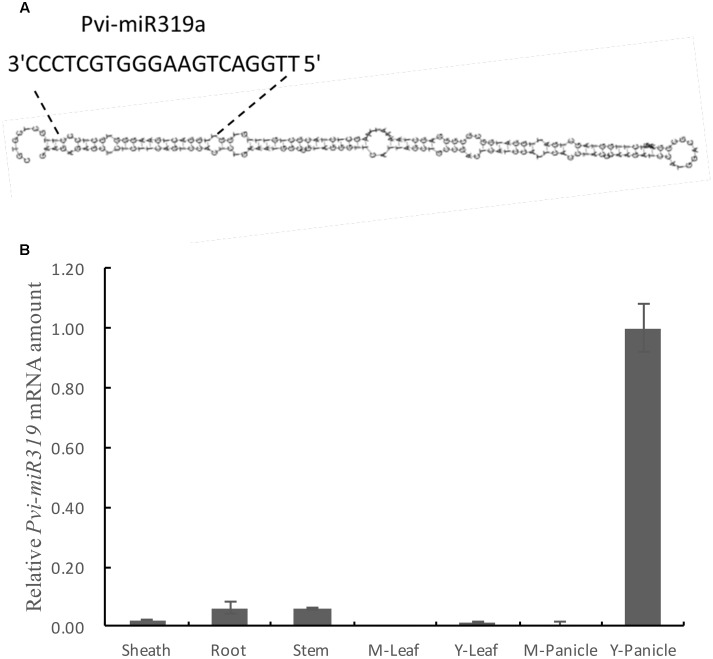
**Predicted stem-loop structure and expression profile analysis of pvi-miR319a in switchgrass.** **(A)** A putative structure diagram of Pvi-miR319a stem-loop area. The enlarged site is complete sequence of mature Pvi-miR319a. **(B)** Expression levels of Pvi-miR319a in different switchgrass tissues were checked by qRT-PCR analysis. Root from 2-week-old seedlings was harvested to be used as experimental materials, while other tissues were collected from switchgrass at heading stage. Rice *ACTIN 1* gene was involved in this experiment as a internal reference.

Considering the close phylogenetic relationship between switchgrass and rice, we hypothesized that the sequence and biological functions of miR319s have conserved in the two species. We could use the known information of the rice miR319 to identify and clone its homologs in switchgrass. Based on the miRNA database^6^, two members (Osa-miR319a and Osa-miR319b) of the rice miR319 family have common mature miRNA sequences (Yang et al., 2013). Using the stem-loop sequences of the rice miR319 precursor genes as a reference, we found an EST (ID: FL985594) with a length of 748 bp from switchgrass (*P. virgatum*) by BLAST search^7^ (**Supplementary Figure S1**). The miR319 sequences from rice and switchgrass showed that the two mature regions were in complete agreement (**Supplementary Figure S2**). Sequence similarity analysis showed that EST has substantial similarity with the *osa-miR319a* precursor gene (**Supplementary Figure S3**). This suggests that the EST of switchgrass contains the miR319a precursor sequence. And the stem-loop structure of this miR319a was found (**Figure 1A** and **Supplementary Figure S4**), So, we named it as *Pvi-MIR319a* (*P. virgatum MIR319a*).

### *Pvi-MIR319a* Expression Analysis

In plants, a long primary miRNA is first transcripted under the guidance of the miRNA gene. It is then processed into pre-miRNA (precursor miRNA) and, finally, into mature miRNA. The mature miRNA recognizes and regulates its target through complementarity to direct RISC-mediated cleavage (Liu and Chen, 2016).

To determine whether *Pvi-MIR319a* plays roles in different plant tissues and developmental stages, we conducted quantitative real-time PCR of *Pvi-MIR319a* transcripts in roots, stems, leaf sheaths, leaves, and panicles. *Pvi-MIR319a* transcript showed a higher expression level in young panicles, indicating that this gene may plays an important role in inflorescence development (**Figure 1B**). This result is consistent with the findings reported by Nag et al. (2009) with regard to the role of miR319a in flower development (Nag et al., 2009).

The expression level of *Pvi-MIR319a* was low in roots, stems, and mature panicles. This is similar to the case in rice, where the expression level of miR319 was high only in young panicles, low in other tissues, and lowest in mature leaves and mature panicles (Yang et al., 2013).

### Generation of Transgenic Rice Plants Overexpressing *Pvi-MIR319a*

**FIGURE 2:**
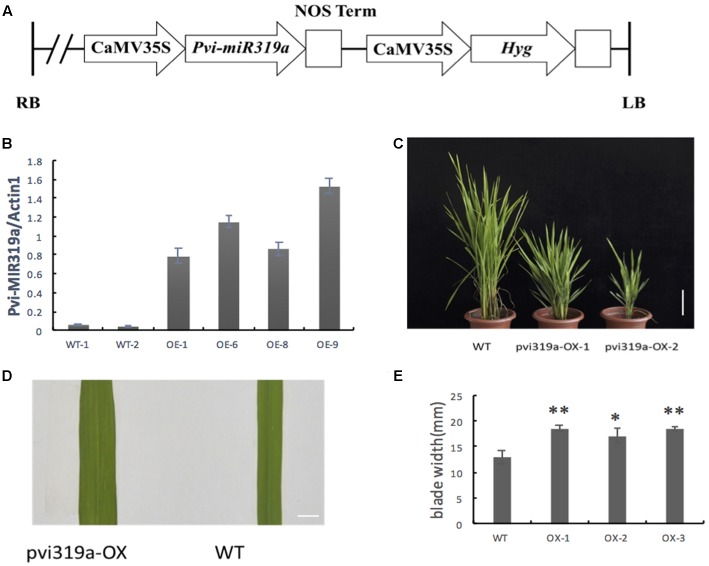
**Identification and phenotypes analysis of the *Pvi-MIR319a*-overexpressing transgenic plants.** **(A)** A schematic of the T-DNA region of the binary construct for rice transformation. **(B)** *Pvi-MIR319a* expression in transgenic lines and wild type rice was detected by qRT-PCR analysis. Transcripts of *Pvi-MIR319a* showed a significantly higher accumulation in transgenic rice plants than that in WT controls. **(C)** Two-month-old transgenic plants showed lower plant height and slower growth compared with the wild-type rice. Bar = 10 cm. **(D)** Comparison blade width between the transgenic plants over-expressed *Pvi-MIR319a* and WT controls. Bar = 10 mm. **(E)** Quantitative measurement and remarkable analysis of average blade width of *Pvi-MIR319a* overexpressing lines and WT control. ^∗^represent a significant difference between transgenic and control plants at *P* < 0.05 by Student’s *t*-test. ^∗∗^represents a remarkable difference between the overexpressing plants and WT control at *P* < 0.01 applying *t*-test method.

To investigate the function of Pvi-MIR319a, *Pvi-MIR319a* gene under the control of 35S promoter, were introduced into rice (**Figure 2A**). Transgenic rice plants overexpressing *Pvi-MIR319a* were obtained to identify whether overexpressing plants had different phenotypes and growth conditions compared with wild-type plants. Positive transgenic plants were identified using RT-PCR method with genomic DNA extracted from transgenic leaves as a template.

A total of 17 independent positive transgenic lines were tested and evaluated. To determine the expression level of *Pvi-MIR319a*, four transgenic lines were analyzed using qRT-PCR. Two wild-type plants were used as controls. The abundance of *Pvi-MIR319a* precursors in transgenic plants was higher than that in control plants (**Figure 2B**), suggesting the *Pvi-MIR319a* fusion plasmids, driven by the 35S promoter, were successfully expressed in rice. It is possible that the ectopic expression of *Pvi-MIR319a* cloned from switchgrass could affect the normal growth and development of transgenic rice plants, which would help in examining the biological function of the switchgrass miRNA using reverse genetic analysis.

### Overexpression of Switchgrass *Pvi-MIR319a* Results in Pleiotropic Phenotype Changes in Transgenic Rice Plants

As a highly conserved ancient miRNA, miR319 plays important roles in plant development (Jones-Rhoades et al., 2006; Yang et al., 2013; Zhou et al., 2013; Zhou and Luo, 2014). High levels of miR319 or downregulation of its target genes leads to a broader leaf phenotype in arabidopsis (Palatnik et al., 2003), and similar results were found in snapdragons, tomatoes, rice, and other plants (Ori et al., 2007; Yang et al., 2013). Although the role of miR319 in leaf morphogenesis is highly conserved, there are several differences in leaf phenotype between dicotyledonous and monocotyledonous plants. Crinkled leaves appear in transgenic dicotyledonous plants ectopically expressing miR319 but not in transgenic monocotyledonous plants. Based on the highly conserved roles of miR319, we hypothesize that *Pvi-MIR319a* may play a similar role in leaf morphogenesis and lead to similar leaf phenotypes in monocotyledonous plants (Yang et al., 2013; Zhou et al., 2013). Overexpression of *Pvi-MIR319a* in rice increased the number of leaf veins and led to broader leaves with a larger leaf area (**Figures 2C–E**). Very similar leaf phenotypes were observed in transgenic rice/bentgrass overexpressing osa-miR319 (Yang et al., 2013; Zhou et al., 2013; Zhou and Luo, 2014). These results suggest that the function of miR319 in leaf morphogenesis is high conversed in grass family (Poaceae).

Analysis of transgenic rice plants (**Figure 2**) constitutively expressing *Pvi-MIR319a* showed that transgenic plants exhibit very distinct phenotypes during the life cycle, including broader and slightly curly leaf blades, shorter plants, and delayed development. These different phenotypes are first observed when transgenic plants initially enter the vegetative growth phase. Measurements were taken from the widest point on the blades at the same position. Statistical analysis showed that leaf blades were significantly wider compared with wild-type (WT) plants and controls. Another important feature is growth retardation, in which overexpressing (OE) lines are shorter than the wild-type. The heading date of transgenic rice plants was also delayed. *Pvi-MIR319a* overexpression transgenic rice plants delayed flowering by about 14–20 days compared with those of wild type lines. We noticed that the similar report was appear in *Arabidopsis thaliana*, overexpression miR319 (*jaw-D* mutants) lead the late flowering in *Arabidopsis thaliana* (Schommer et al., 2008). Those results suggest that the function of miR319 in flowering may conservative, and it deserves study deeper in the future.

### Switchgrass *Pvi-MIR319a* Regulates Expression of Putative Target Genes in Transgenic Rice Plants

**FIGURE 3:**
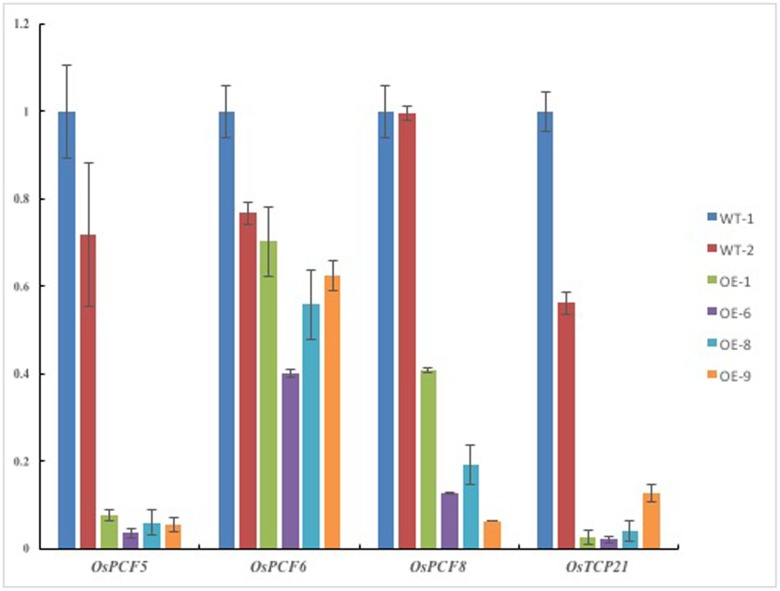
**Expression levels of putative targets were analysed in *Pvi-MIR319a*-overexpressing rice plants and wild types by real-time quantitative PCR.** Compared with WT controls, expression of three predicted target genes were decreased in different degree in transgenic lines. Rice *ACTIN 1* (*LOC_Os10g36650*) is used as a reference gene.

We performed a series of qRT-PCR tests to analyze the transcript levels of five TCP genesin *Pvi-MIR319a*-overexpressing rice. Expression analysis showed that the levels of the four genes (*OsPCF5*, *OsPCF6*, *OsPCF8*, and *OsTCP21*) were downregulated compared with those of WT, in which *OsPCF5*, *OsPCF8*, and *OsTCP21* were significantly reduced (**Figure 3**). The obvious decrease in the expression of the predicted targets was similar to that observed in osa-miR319a-overexpressing rice (Yang et al., 2013). We hypothesize that mRNAs from the predicted target genes may be directly degraded by *Pvi-MIR319a* due to the high degree of homology between *Pvi-MIR319a* and *Osa-miR319a*. Based on the abnormal leaf phenotype of transgenic plants and the early functional studies on TCP transcription factors, we think that the continuous expression of *Pvi-MIR319a* leads to a series of pleiotropic phenotypic changes in transgenic rice plants due to the negative regulation of its possible target genes. In this study, higher levels of target transcripts enabled leaves to maintain normal growth, whereas target mRNAs were cleaved by overexpressing *Pvi-MIR319a*, thereby leading to broad leaves and abnormal developmental patterns. It has been reported that miR319-targeted TCPs are involved in leaf development in eudicot species, such as arabidopsis and tomato (Nag et al., 2009; Martín-Trillo and Cubas, 2010). However, the biological functions of both *PvPCF5* and *OsPCF5* genes are largely unknown. It is worthy to do further study. Taken together, the results imply that *Pvi-MIR319a* and its targets play critical roles in plant development.

### *PvPCF5* is a miR319a Target Gene in Switchgrass

The gene sequence of *PvPCF5* was cloned and identified. Sequence analysis showed that *PvPCF5* encodes a protein with a TCP DNA-binding domain at the N-terminus, and the complete domain was obtained by 5′ RACE assay. We proposed that PvPCF5, with a length of 444 amino acids and a molecular mass of 46.4 kilodaltons, is a transcription factor belonging to the TCP family (**Supplementary Figure S5**).

**FIGURE 4:**
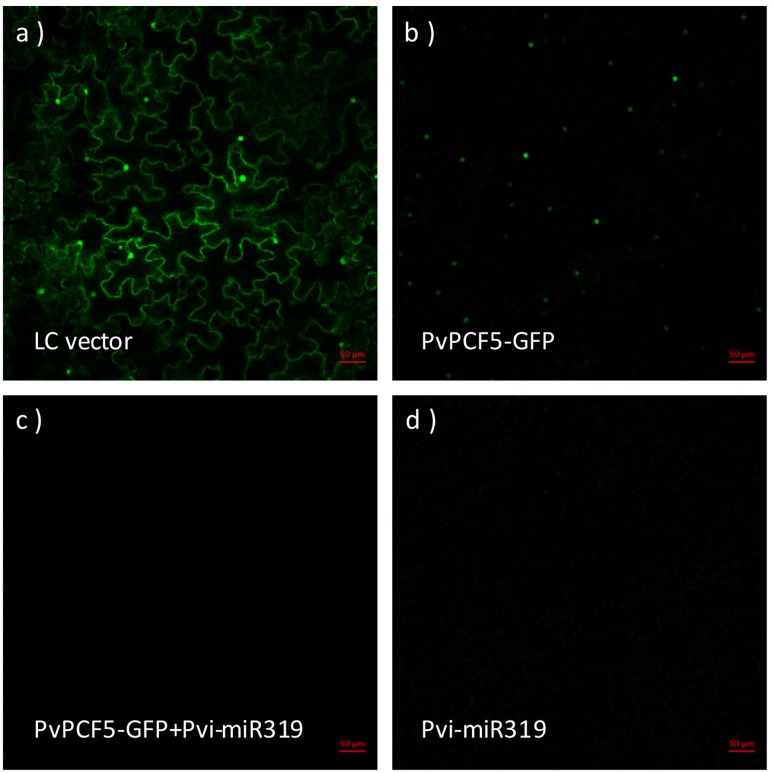
***PvPCF5* is a miR319a target supported by transient expression assay in *Nicotiana benthamiana*.** *N. benthamiana* leaves were injected with *Agrobacterium*, respectively, carrying the empty vector (LC) **(a)**; 35S::PvPCF-GFP **(b)**; 35S::PvPCF-GFP plus 35S::*Pvi-MIR319a* **(c)**; and 35S::*Pvi-MIR319a* **(d)**. GFP green fluorescent signals were observed by using a confocal microscope. Bar = 50 μm.

To determine that *PvPCF5* is an important target of *Pvi-MIR319a*, a rapid *Agrobacterium*-mediated transient expression assay was performed in *N. benthamiana.* Fusion plasmids (35S::PvPCF5-GFP and 35S::*Pvi-MIR319a*) were constructed and introduced into *Agrobacterium* strain EH105 with a control vector. Transient transformation was carried out by co-injection into tobacco leaves. After 48 h of recovery growth, a GFP signal was observed by the UV visualization of green fluorescence under a confocal microscope. The results showed that only tobacco leaves injected with single 35S::PvPCF5-GFP constructs or empty vectors generated GFP fluorescence, whereas co-expression with 35S::*Pvi-MIR319a* led to a notably weaker GFP signal (**Figure 4**). This shows that the *PvPCF5* levels were markedly decreased when co-expressed with 35S::*Pvi-MIR319a*. These results provide direct evidence that *PvPCF5* is a target of *Pvi-MIR319a* and that *Pvi-MIR319a* can regulate the expression of *PvPCF5*, leading to a significantly lower expression level by direct cleavage (**Supplementary Figure S6**). Additionally, **Figure 3** shows that the GFP signal was localized around the cell nucleus in tobacco leaves, indicating that PvPCF5 is a nuclear-localized protein.

### Expression Profile of *PvPCF5*

**FIGURE 5:**
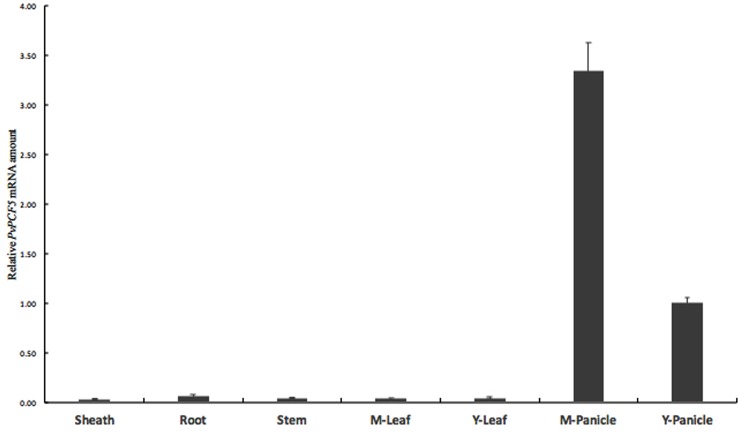
**Expression pattern of *PvPCF5* gene in various organs by qRT-PCR assay.** Switchgrass organs include sheath, root, stem, mature leaf, mature panicle, and young panicle were harvested from plants at heading stage. Young leaves were collected before tillering stage. Young panicles were harvested at less than 5 cm.

*PvPCF5* was identified as one of the target gene of *Pvi-MIR319a* in switchgrass. The abundance of this target gene in various tissues was examined by quantitative real-time PCR (**Figure 5**). *PvPCF5* is highly expressed in mature panicles, but it is weakly expressed in young panicles, leaf sheaths, stems, and roots, with the lowest expression levels in leaves. The expression level of *PvPCF5* had a relatively negative correlation with the expression of *Pvi-MIR319a*, at least in young and mature panicles.

### PvPCF5 Encodes a TCP Transcription Factor

**FIGURE 6:**
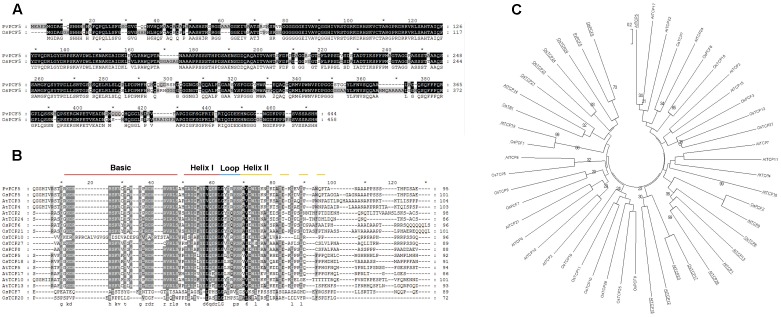
**PvPCF5 is a TCP transcript factor and has the highest homology with rice OsPCF5.** **(A)** Amino acid sequence alignment of switchgrass PvPCF5 and rice OsPCF5. **(B)** Parts of PvPCF5 protein sequence was comparatively analyzed with other TCPs selected from CIN subfamily. PvPCF5 and other TCP members are conserved in TCP DNA-binging domain. bHLH structure position was marked in this figure. **(C)** The phylogenetic relationship between PvPCF5 and other TCP transcription factor subfamily members from rice and arabidopsis. The phylogenetic tree was constructed by using MEGA 5.0 software. The number in the diagram represent the bootstrap value from 500 replicates.

Based on the similarity of the amino acid sequences of the TCP domain region, TCP subfamily proteins are classified into three subgroups: PCF, CIN, and CYC/TB1 (Martín-Trillo and Cubas, 2010). Almost all TCP family members with miR319-binding sites belong to the CIN subgroup, including OsPCF5 (Yang et al., 2013). Previous studies also showed that most CIN subfamily members are involved in leaf development in various plant species. Sequence alignment analysis of PvPCF5 protein from switchgrass and OsPCF5 cloned from rice was performed to determine their homology (**Figure 6**). Results revealed that PvPCF5 had high homology to OsPCF5. To investigate the evolutionary relationships between PvPCF5 and other TCP subfamily TFs from rice and arabidopsis, a series of related TCP transcription factors, including 26 from arabidopsis and 25 from rice, were collected for phylogenetic analysis. The phylogenetic tree (**Figure 6C**) indicated that PvPCF5, targeted by *Pvi-MIR319a*, was clustered in the CIN subfamily, and AtTCP4 was most closely related to PvPCF5. The OsPCF5 transcription factor is important in rice leaf development, and there is high conservation between PvPCF5 and OsPCF5 indicating that PvPCF5 may be primarily involved in leaf morphological development (Yang et al., 2013). Multiple alignments among the 51 TCP proteins confirmed the presence of the TCP domain in PvPCF5 near the N-terminus. However, there were major differences in other areas of the TCP proteins, suggesting that these differences may be due to protein functional specificity.

**FIGURE 7:**
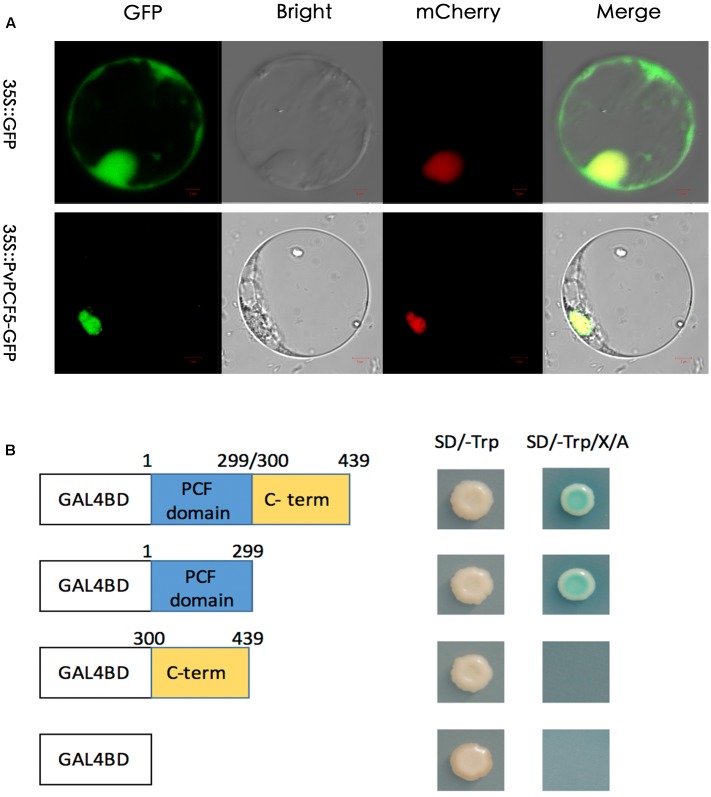
**Subcellular localization and transcriptional activation analysis of PvPCF5.** **(A)** Subcellular localization of PvPCF5 was performed in switchgrass protoplasts. Confocal images showing the PvPCF5-GFP fusion protein driven by the 35S promoter was specifically expressed in the protoplasts. 35S::GFP was used as a control. Bar = 10 μm. **(B)** Transactivation activity of the PvPCF5 protein in yeast. The full-length, N-terminal region, C-terminal region of the *PvPCF5* were fused into DNA sequences containing a GAL4 DNA-binding domain with the negative control plastid (pGBKT7). These four kinds of constructs were expressed in the yeast strain Y2HGold. The transformants were streaked on the SD/-trp and SD/-trp/A/X-α-gel plates, plates were cultured for 2–4 days at 30°C to check a possible transcriptional activation function of PvPCF5.

To investigate the subcellular localization of the PvPCF5 protein, a 35S::PvPCF5-GFP fusion and a GFP control were obtained and transformed into prepared switchgrass protoplast cells. The PvPCF5-GFP gene was driven by the CaMA35S expression promoter (**Figure 7A**). Based on the observed fluorescence signal, 35S:: PvPCF5-GFP was targeted to the cell nucleus, whereas the fluorescence signal of the negative control was observed over the entire protoplast. These results show that PvPCF5 is a nuclear-localized protein, which is a strong indicator of a transcription factor.

We use a yeast system to determined whether PvPCF5 has transactivation activity (**Figure 7B**). The pGBKT7-PvPCF5, pGBKT7-N (aa 1–299), or pGBKT7-C (aa 300–439) fusion constructs or an empty BD vector (pGBKT7) were transformed into the Y2HGold yeast strain. As shown in **Figure 7B**, all yeast cells showed good growth on the SD/-Trp plates. However, only cells containing the pGBKT7-PvPCF5 and pGBKT7-N (aa 1–299) constructs could form colonies on SD/-Trp/A/X-α-gel plates. In the presence of abscisic acid and X-α-gel, yeast colonies turned blue, indicating that other reporter genes, such as AUR1-C and MEL1, were activated. These phenomena illustrate that the PvPCF5 has transcription activation activity.

## Conclusion

We cloned *Pvi-MIR319a* from switchgrass and employed reverse genetics to investigate its function. Overexpression of *Pvi-MIR319a* in transgenic rice increased leaf width, reduced plant height, and delayed heading time. It suggests that *Pvi-MIR319a* and its target genes may be used as potential genetic regulators for future switchgrass genetic improvement. As a model energy plant, the high effective *Agrobacterium*-mediated transformation systems of switchgrass has been established (Liu et al., 2015). Next, we plan to express the *Pvi-MIR319a* and *PvPCF5* into switchgrass itself, with its own promoters, constitutive promoters or tissue-specific expression promoters, respectively. The gene editing approaches for*Pvi-MIR319a* and *PvPCF5* by CRISPR/CAS9 would also be considered. And then we will screen the elite agronomic traits from the transgenic lines to improve the quality of switchgrass.

## Author Contributions

Designed the experiments: GS, LH, XL, and DL. Preformed the experiments: QX, YZ, XL, DY, BF, and JT. Analyzed the data: YZ, XL, LZ, DL, and JT. Wrote the paper: YZ, QX, DL, and XL.

## Conflict of Interest Statement

The authors declare that the research was conducted in the absence of any commercial or financial relationships that could be construed as a potential conflict of interest.

## Footnotes

1 http://www.mirbase.org

2 https://www.arabidopsis.org/index.jsp

3 http://rice.plantbiology.msu.edu/

4 http://www.zeiss.com

5 http://www.takara.com.cn/ProductShow.aspx?m=20141220165734077123&productID=20141227185621610423

6 http://www.mirbase.org

7 https://blast.ncbi.nlm.nih.gov/Blast.cgi

## Supplementary Material

**FIGURE S1 | The information of *Pvi-MIR319* sequence.**

**FIGURE S2 | Alignment of mature miR319s.**

**FIGURE S3 | Alignment of the stem-loop sequences.**

**FIGURE S4 | Phylogenetic tree analysis of mature miR319a.**

**FIGURE S5 | The structure and sequences of *PvPCF5*.**

**FIGURE S6 | The target site between *Pvi-MIR319a* and *PvPCF5*.**

**TABLE S1 | The information of primers.**
