# Supplementary figures and images for: Identification and Characterization of microRNA319a and Its Putative Target Gene, PvPCF5, in the Bioenergy Grass Switchgrass (Panicum virgatum)
Source: Front Plant Sci. 2017 Mar 30;8:396. doi: 10.3389/fpls.2017.00396 (PMC5371612; doi:10.3389/fpls.2017.00396)

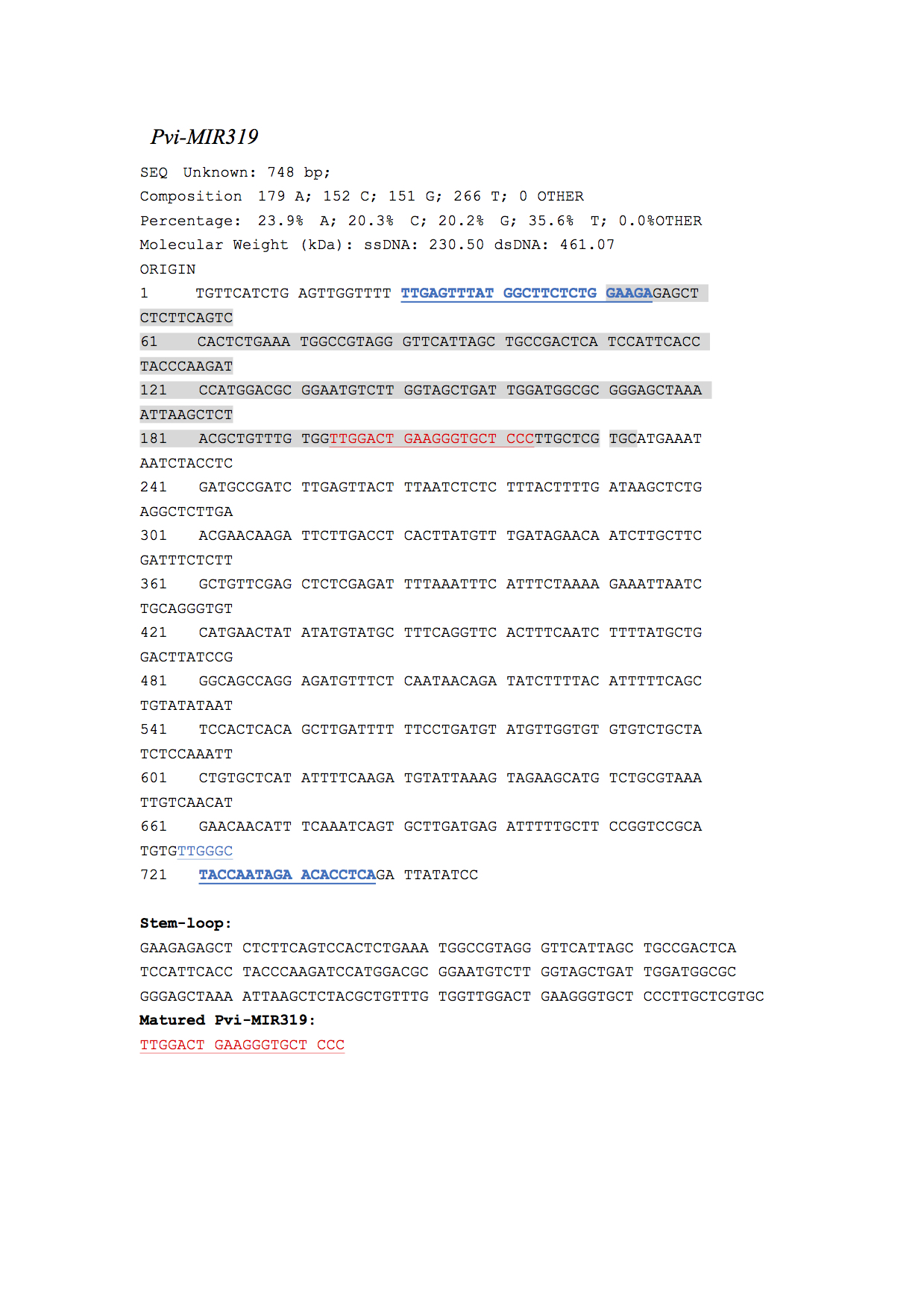

Supplement: Supplementary file 1 [file Image_1.JPEG]

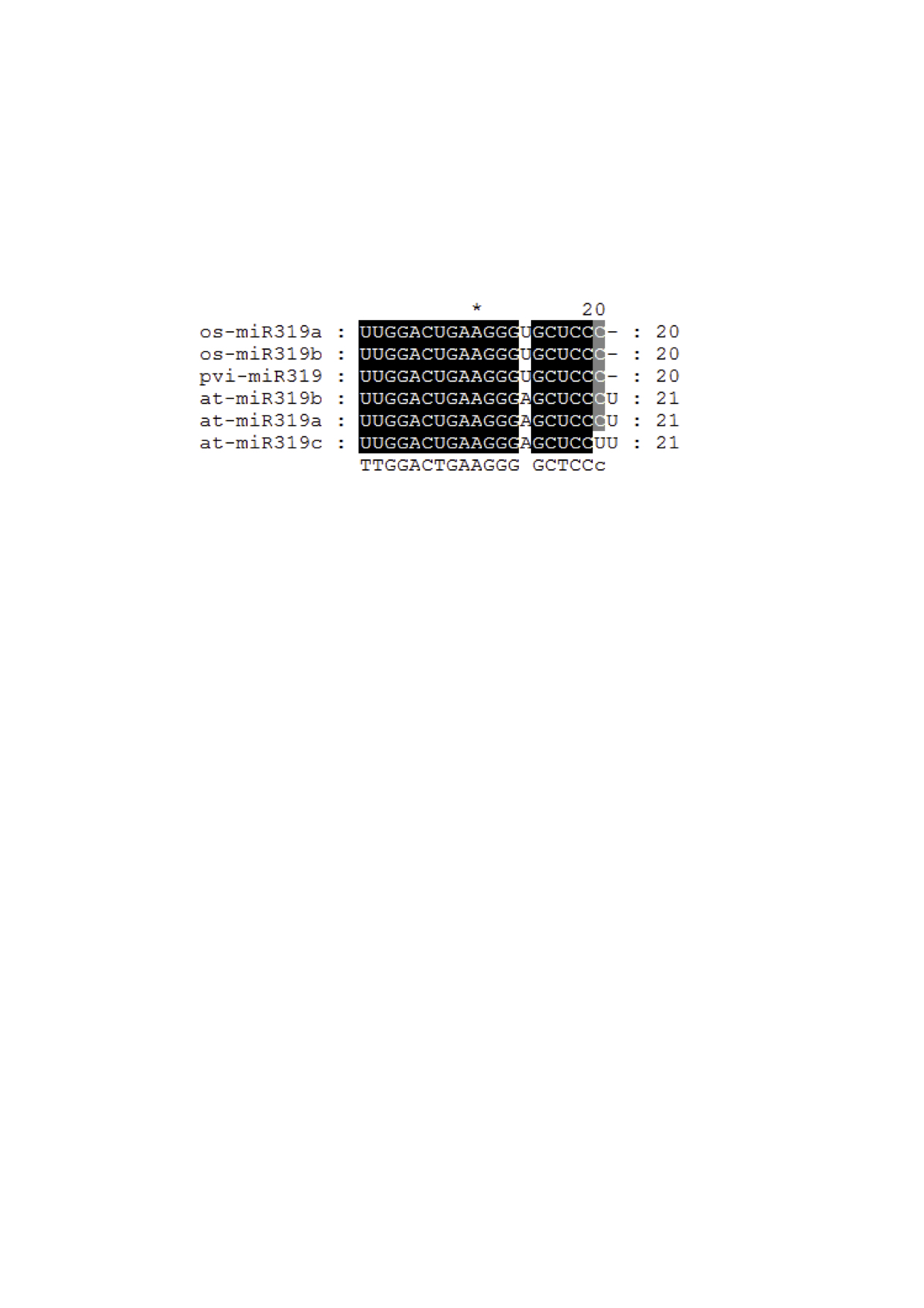

Supplement: Supplementary file 2 [file Image_2.JPEG]

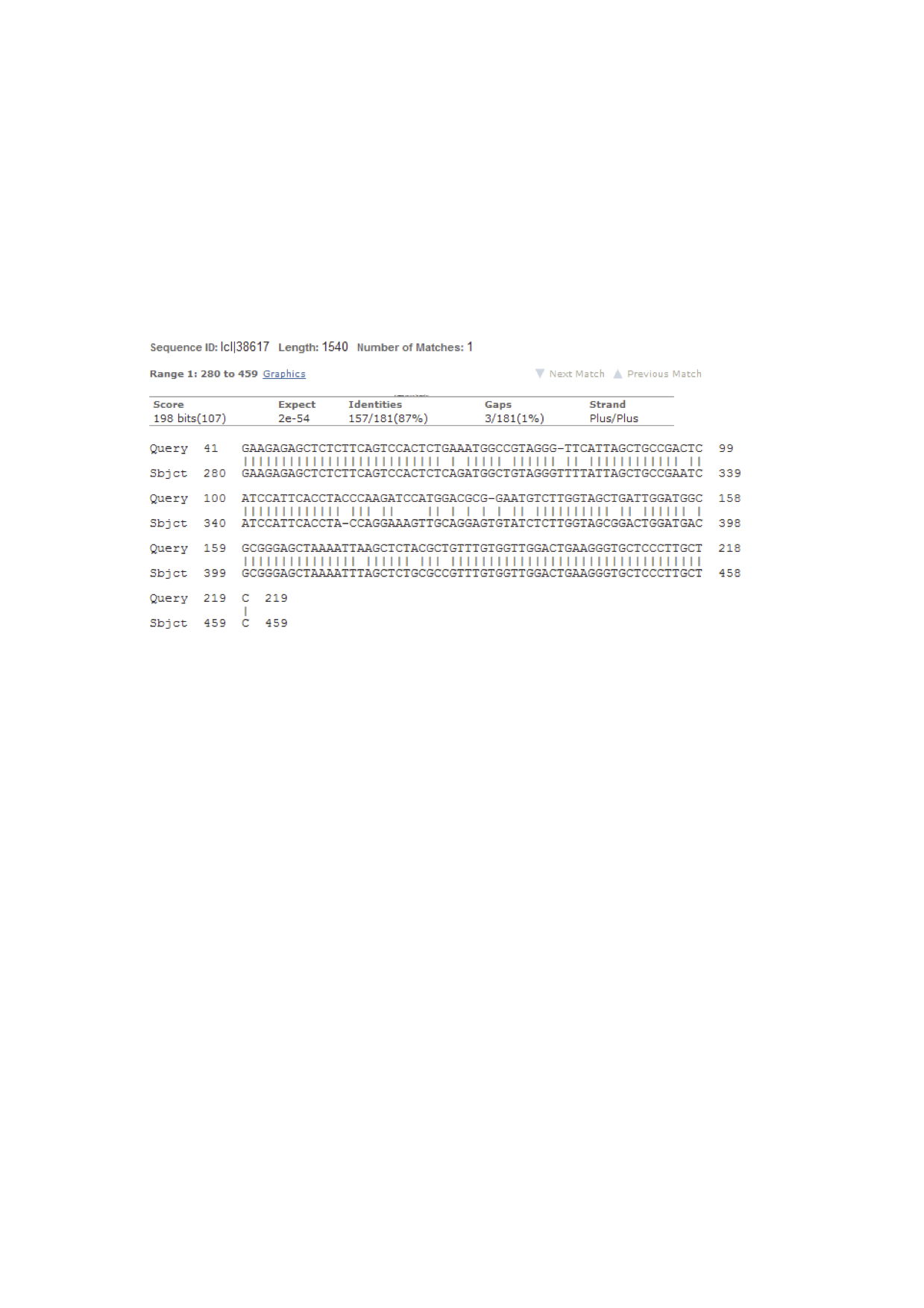

Supplement: Supplementary file 3 [file Image_3.JPEG]

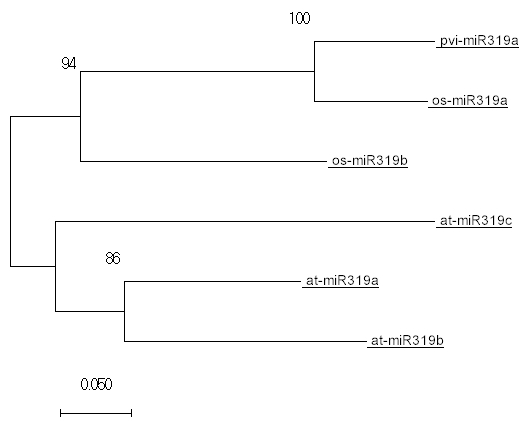

Supplement: Supplementary file 4 [file Image_4.JPEG]

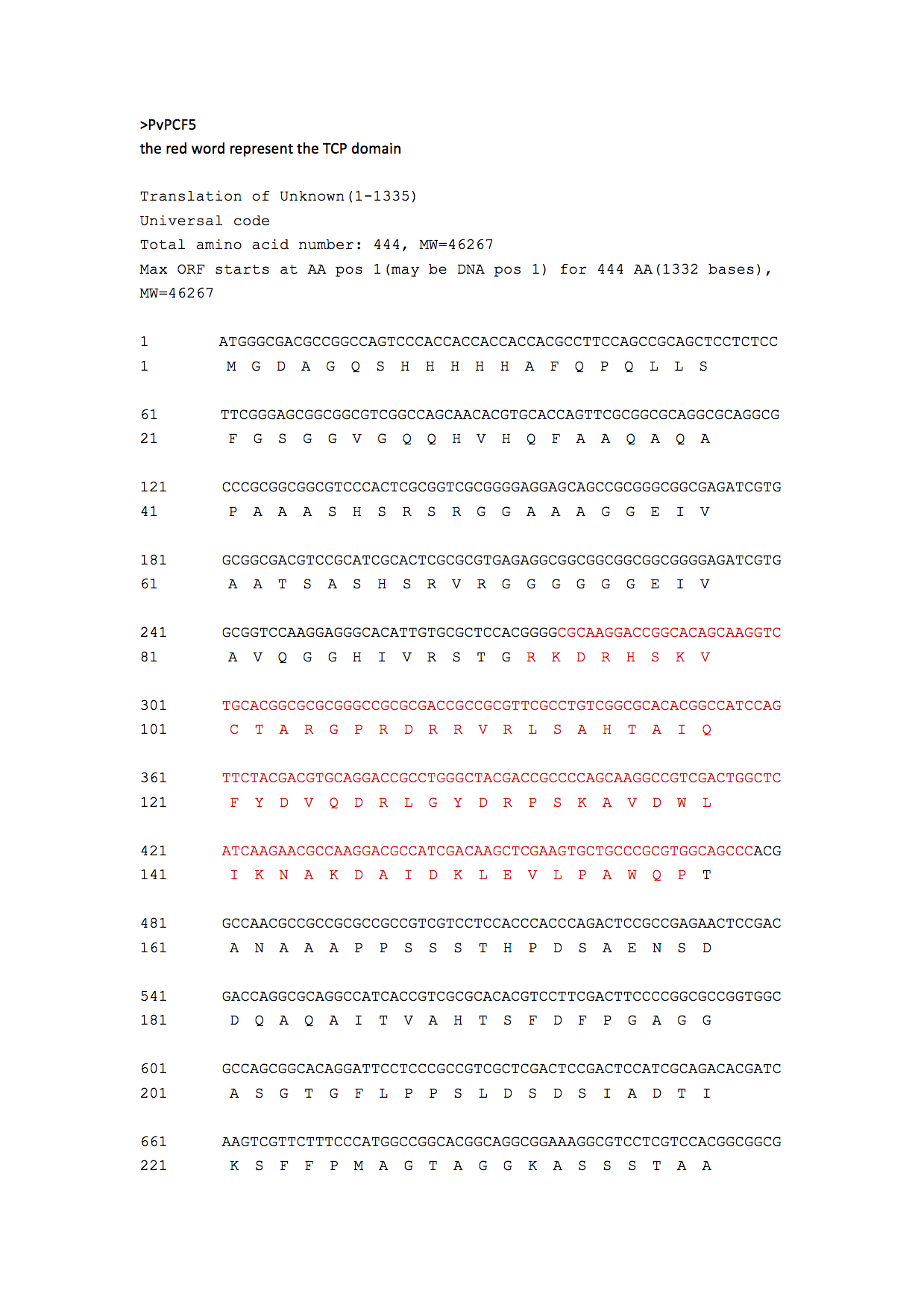

Supplement: Supplementary file 5 [file Image_5.JPEG]

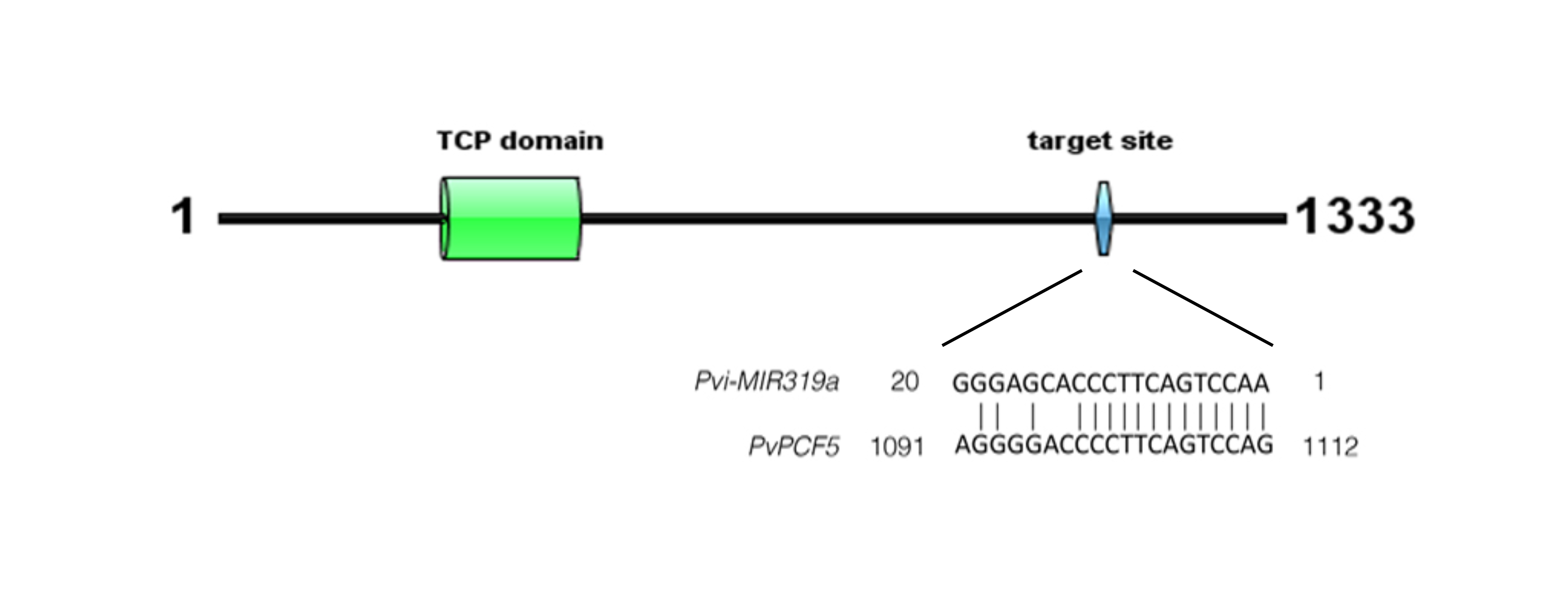

Supplement: Supplementary file 6 [file Image_6.JPEG]
