# Supplementary material for: Identification and Characterization of microRNA319a and Its Putative Target Gene, PvPCF5, in the Bioenergy Grass Switchgrass (Panicum virgatum)
Source: Front Plant Sci. 2017 Mar 30;8:396. doi: 10.3389/fpls.2017.00396 (PMC5371612; doi:10.3389/fpls.2017.00396)
Supplement: Supplementary file 7 [file Table_1.DOCX]

| Pvi-MIR319a | Forward: 5’- TCTAGATTGAGTTTATGGCTTCTCTGGAAGA -3’ |  |
| --- | --- | --- |
|  | Reverse: 5’- GTCGACTGAGGTGTTCTATTGGTAGCCCAA -3’ |  |
| Pvi-MIR319a | Forward: 5’- **AAGCTT**TGAGTTTAT GGCTTCTCTG GAAGA -3’ |  |
|  | Reverse: 5’- **GAATTC**TGAGGTGTTC TATTGGTAGC CCAA -3’ |  |
| PvPCF5 | Forward: 5’- TGAATTCATGGGCGACG CCGGCCAGTC -3’ |  |
|  | Reverse: 5’- TGGATCCGTGGTGAGAA GCCGAGGACA -3’ |  |
| PvPCF5(1-299aa) | Forward: 5’- C ATG GAG GCC GAATTC ATGGGCGACGCCGGCCAGTCC-3’ |  |
|  | Reverse: 5’- GC AGGTCGAC GGATCC CGCCTGCTGC GCGGAGCCAT TG -3’ |  |
| PvPCF5(300-439aa) | Forward: 5’- C ATG GAG GCC GAATTC CTCTTCCCCGGCG CCGCCAGC -3’ |  |
|  | Reverse: 5’- GC AGGTCGAC GGATCC TCAGTGGTGAGAAGCCGAGGAC -3’ |  |
| PvPCF5(1-439aa) | Forward: 5’- C ATG GAG GCC GAATTC ATGGGCGACGCCGGCCAGTCC-3’ |  |
|  | Reverse: 5’- GC AGGTCGAC GGATCC TCAGTGGTGAGAAGCCGAGGAC -3’ |  |
| Pvi-MIR319a | Forward: 5’- CTGAGGCTCTTGAACGAACA -3’ |  |
|  | Reverse: 5’- GTTCATGACACCCTGCAGAT -3’ |  |
| PvPCF5 | Forward: 5’- CCATCCAGTTCTACGACGTG -3’ |  |
|  | Reverse: 5’- GCACTTCGAGCTTGTCGAT -3’ |  |
| OsPCF5 | Forward: 5’- CTCATCAAGAACGCCAAGGAC -3’ | (Yang et al., 2013) |
|  | Reverse: 5’- GAGGGCTGGTTACTGGACT -3’ |  |
| OsPCF6 | Forward: 5’- ATCGATCCCAGCAACGCAT -3’ | (Yang et al., 2013) |
|  | Reverse: 5’- GCAGAACGATGACCCAGCTAAA -3’ |  |
| OsPCF8 | Forward: 5’- ATGTCTGGTGTTGTGCCACAAG -3’ | (Yang et al., 2013) |
|  | Reverse: 5’- GAGAAATGGAAATCATGAGCCG -3’ |  |
| OsTCP21 | Forward: 5’- GGAGATGACGCACTACTCGT -3’ | (Yang et al., 2013) |
|  | Reverse: 5’- ATGGCTGGAGAGGTGAGACT -3’ |  |
| OsACTIN1 | Forward: 5’- TCCATCTTGGCATCTCTCAG -3’ | (Yang et al., 2013) |
|  | Reverse: 5’- GTACCCGCATCAGGCATCTG -3’ |  |
| PviUBQ | Forward: 5’- TTCGTGGTGGCCAGTAAG -3’ |  |
|  | Reverse: 5’- AGAGACCAGAAGACCCAGGTACAG -3’ |  |

Yang, C., Li, D., Mao, D., Liu, X., Ji, C., Li, X., et al. (2013). Overexpression of microRNA319 impacts leaf morphogenesis and leads to enhanced cold tolerance in rice (Oryza sativa L.). *Plant Cell & Environment* 36(12)**,** 2207–2218.
